# IFN-λ: A New Inducer of Local Immunity against Cancer and Infections

**DOI:** 10.3389/fimmu.2016.00598

**Published:** 2016-12-15

**Authors:** Ahmed Lasfar, Andrew Zloza, Andrew de la Torre, Karine A. Cohen-Solal

**Affiliations:** ^1^Department of Pharmacology and Toxicology, Ernest Mario School of Pharmacy, Rutgers, The State University of New Jersey, Piscataway, NJ, USA; ^2^Rutgers Cancer Institute of New Jersey, New Brunswick, NJ, USA; ^3^Section of Surgical Oncology Research, Department of Surgery, Rutgers Robert Wood Johnson Medical School, Rutgers, The State University of New Jersey, New Brunswick, NJ, USA; ^4^Department of Surgery, New Jersey Medical School, Rutgers, The State University of New Jersey, Newark, NJ, USA; ^5^St Joseph’s Medical Center, Paterson, NJ, USA

**Keywords:** IFN-λ, mucosal immunity, viral infections, immunotherapy of cancer, inflammation, NK cells

## Abstract

IFN-λ is the newly established type III IFN with unique immunomodulatory functions. In contrast to the IFN-α/β family and to some extent IFN-γ, IFN-λ is apparently acting in specific areas of the body to activate resident immune cells and induces a local immunity, instrumental in preventing particular infections and also keeping transformed cells under control. Mucosal areas of lung and gastrointestinal tracts are now under scrutiny to elucidate the immune mechanisms triggered by IFN-λ and leading to viral protection. New evidence also indicates the crucial role of IFN-λ in promoting innate immunity in solid cancer models. Based on its unique biological activities among the IFN system, new immunotherapeutic approaches are now emerging for the treatment of cancer, infection, and autoimmune diseases. In the present review, we highlight the recent advances of IFN-λ immunomodulatory functions. We also discuss the perspectives of IFN-λ as a therapeutic agent.

## Introduction

Human IFN-λs are represented by four functional and highly homologous subtypes IFN-λ1, IFN-λ2, IFN-λ3, and IFN-λ4 (1, 2). In contrast to the other IFN-λ subtypes, IFN-λ4 is selectively expressed in the human population and weakly released by IFN-producing cells (3). However, all the four IFN-λ protein subtypes are clustered on chromosome 19 and are grouped in a new IFN family, called type III IFN, distinct from type I and type II IFNs, respectively, representing the classical IFN-α/β family and IFN-γ. In mice, only two functional genes located on chromosome 7 and encoding IFN-λ2 and IFN-λ3 have been characterized (4). In contrast to its human counterpart, the murine IFN-λ1 gene ortholog is a pseudogene as reported in several mice strains. However, we did not find yet a corresponding IFN-λ4 in mice. Type III IFNs use a unique receptor, the IFN-λ receptor, and induces similar JAK–STAT signaling pathway as type I IFNs (5, 6). Although it has been well established that in addition to JAK1, TYK2 is crucial in mediating the activity of both type I and type III IFN, new evidence in patients with a defect in TYK2 shows an impaired response for type I IFN only (7), suggesting a Tyk2-independent signaling for type III IFN. Upstream cell signaling is quite distinct between type I and type III IFN. Type I IFN interacts with a receptor formed by IFNAR1 and IFNAR2 (8). However, type III IFNs bind to the specific receptor chain IFN-λR1, and IL-10R2, a receptor subunit shared by IL-10 cytokine family members IL-10, IL-22, and IL-26 (6). In contrast to type I and type II IFN receptors, the unique type III IFN receptor for IFN-λ, IFN-λR1 is not ubiquitously expressed (5, 9), suggesting that IFN-λ may eradicate specific viral infections and also elicit a more local immunity against pathogens and cancers. This has important consequences for therapeutic targeting (10, 11).

In addition to its restricted interaction mainly with epithelial cells (EC), IFN-λ may also induce cell signaling that differs to some extend from IFN-α/β signaling. Currently, it has been established that the antiviral patterns of IFN-λ are quite distinct from those of IFN-α (10). In oncology and autoimmune diseases, the role of IFN-λ seems also to differ in many aspects from IFN-α (11).

Furthermore, in contrast with type I, type III IFN is prone to a particular genetic reactivation or deactivation in the human population as illustrated with IFN-λ4 and related genetic polymorphism (3). Tremendous research efforts are still ongoing to understand the impact of this genetic aspect of type III IFN on the prevalence of diseases, particularly hepatitis. IFN-λ4 has been linked with the failure to clear hepatitis C virus (HCV) infection and decreased response of HCV patients to IFN-α therapy (12, 13). IFN-λ4 can be produced only by people who carry the IFN-λ4–ΔG allele (rs368234815), known for predicting HCV clearance (3, 14). The inherited IFN-λ4–ΔG allele is the main variant in Africans, while the minor variant is found in Asian people (15). Therefore, a negative genetic selection for IFN-λ4–ΔG allele could be driven by infectious agents, including HCV (16).

As highlighted in many reviews, the activity of IFN-λ is highly prominent in EC in comparison with other cell types (1, 3, 5, 9–11, 17–24). However, the significance of the restricted action of IFN-λ remains elusive. We still have to understand the role of this specific interaction of IFN-λ on the protection of epithelial surfaces from exposure to pathogenic microbes and the development of carcinomas. The goal of this review is not an exhaustive description of the IFN-λ biology, which has been abundantly reported in many important reviews (5, 10, 20, 22–25). We have been focusing our review on the potential links between the prominent activity of IFN-λ on EC and its associated immunity against viral infections and cancer.

## IFN-λ and the Epithelium Tract Defense against Viral Infections

**Figure 1:**
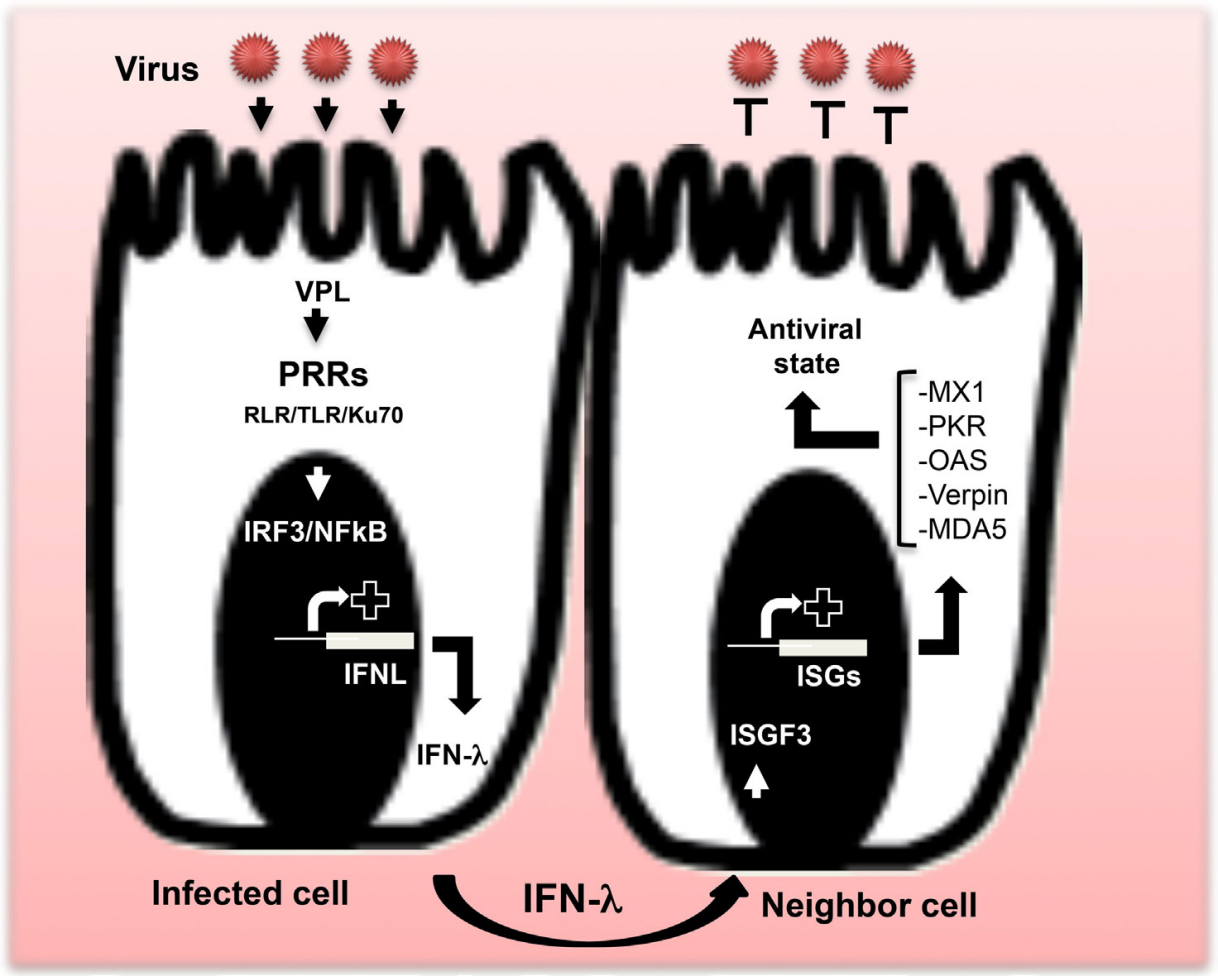
**Production of IFN-λ and establishment of the antiviral state**. After virus attack, IFN-λ genes are induced. When viral particle ligands (VPL) are sensed by pattern recognition receptors (PRRs), particular transcription factors, mainly IRF-3 and NF-κB, are induced to allow IFN-λ gene expression and subsequent release of IFN-λ proteins outside the infected cell. Dependent on the type of virus, various PRRs are involved, including members of the RIG-I-like receptor and toll-like receptor families, as well as the DNA sensor Ku70. Released IFN-λ induces an antiviral state in neighboring cells *via* the induction of interferon-stimulated gene factor leading to the expression of specific antiviral interferon-stimulated genes (ISGs), including myxovirus resistance 1, protein kinase R, melanoma differentiation-associated protein 5, verpin, and 2′-5′-oligoadenylate synthetase. Products of those antiviral ISGs inhibit virus replication and provide an antiviral state to cells.

Accumulating evidence strongly suggests that IFN-λ plays a major role in providing the frontline defense for the epithelium against viruses. The epithelium is formed by closely packed EC with practically no intercellular spaces. However, EC are not isolated from immune cells. Respiratory, urogenital, and gastrointestinal (GI) tracts forming the major mucosal areas in the body show complex association between the epithelium and the immune cells in variable proportions, endowing mucosal surfaces with a particular immunity against the harmful environment (26). By lining mucosal surfaces, EC are under continuous attack by viruses. The first response of cells infected with virus is the release of IFN. The released IFN provides neighboring healthy cells an antiviral state, allowing them to stop viral spreading (Figure 1). However, clearance of infected cells requires immune cells intervention. Both type I and type III IFNs are expressed by host cells in response to viral infection. However, depending on the site of virus attack, host cells exhibit differential expression of type I and type III IFNs (5, 25, 27). The mechanisms leading to the induction of IFN expression, the establishment of antiviral state, and the clearance of infected cells are well documented particularly for type I IFN (10). In contrast, we are still striving to understand the role of IFN-λ-regulated antiviral mechanisms in mucosal surfaces on which increasing reports indicate a critical role of type III IFN.

## Role of IFN-λ in the Control of Viral Infections of the Respiratory Tract

Respiratory tract infections by viruses are common and mainly concern the sinus, the throat, and the lungs. In comparison with IFN-α, IFN-λ is predominantly induced by respiratory viruses (28–31). Currently, IFN-λ is designated as a therapeutic candidate against the influenza A virus (IAV) infection (32, 33). In infants hospitalized for respiratory syncytial virus (RSV)- or human rhinovirus (HRV)-associated bronchiolitis, RSV elicited higher levels of IFN-λ subtypes when compared with HRV (34).

It has been postulated that in order to increase infection, respiratory viruses can also suppress IFN-λ antiviral response. Influenza virus- and RV-induced epidermal growth factor receptor activation has been found to suppress IFN regulatory factor (IRF)-1-induced IFN-λ production and increased viral infection (35). NS1 and NS2 proteins of the human RSV also inhibit IFN-λ production, occurring *via* IFN regulatory factor (IRF)-3, NF-κB, and proinflammatory cytokines suppression (36, 37). More recently, it has been reported that excessive expression of IFN-λ in the lung during IAV infection is associated with a suppression of IFN-λ signaling by SOCS-1 (31). The authors suggested that the suppression of cytokine signaling by virus-induced SOCS-1 leads to an adaptive increase in IFN-λ production by the host to protect cells against viral infection. This increase of IFN-λ production further induces the expression of SOCS-1 at late stage of infection, which in turn, inhibits the activation of JAK–STAT signaling. Finally, this vicious cycle results in excessive production of IFN-λ and impaired antiviral activity.

One of the main concerns about viral lung infections such as the one caused by IAV is the subsequent inflammation. Although IFN-α is highly efficient in suppressing IAV, in contrast with IFN-λ, it exacerbates the inflammation by overstimulating the immune system and driving immunopathology (32). Therefore, in agreement with its weak targeted actions on immune cells surrounding infected EC, IFN-λ may constitute the treatment of choice in viral infections associated with inflammation (11). In favor of IFN-λ as therapeutic option for viral infection associated with inflammation, we can mention early studies on asthma, showing a deficiency in IFN-λ (38) and the role of IFN-λ treatment in suppressing respiratory viral infections and allergic airway inflammation (39). However, other immune mechanisms could also occur after IFN-λ treatment. It has been strongly suggested that by upregulating indoleamine 2,3-dioxygenase during influenza virus infection, IFN-λ may induce an immune suppression (40).

## Role of IFN-λ in the Control of Viral Infections of the Gastrointestinal Tract

Currently, several studies indicate that IFN-λ plays a predominant role in controlling viral infections of the GI tract (5, 10, 41, 42). In response to viral infections, IFN-λ is highly produced by intestinal EC and induces a strong antiviral response (27). However, recent studies show that this strong effect of IFN-λ resulted from a synergistic effect with IL-22 (43). ZEB1 has been shown to play a role in the activation of IFN-λ gene expression at the transcriptional level, in addition to IRF-3 and NF-κB (10, 44). Interestingly, the role of IFN-λ in controlling viral infections of the GI tract cannot be compensated by IFN-α/β in suckling mice (25).

**Figure 2:**
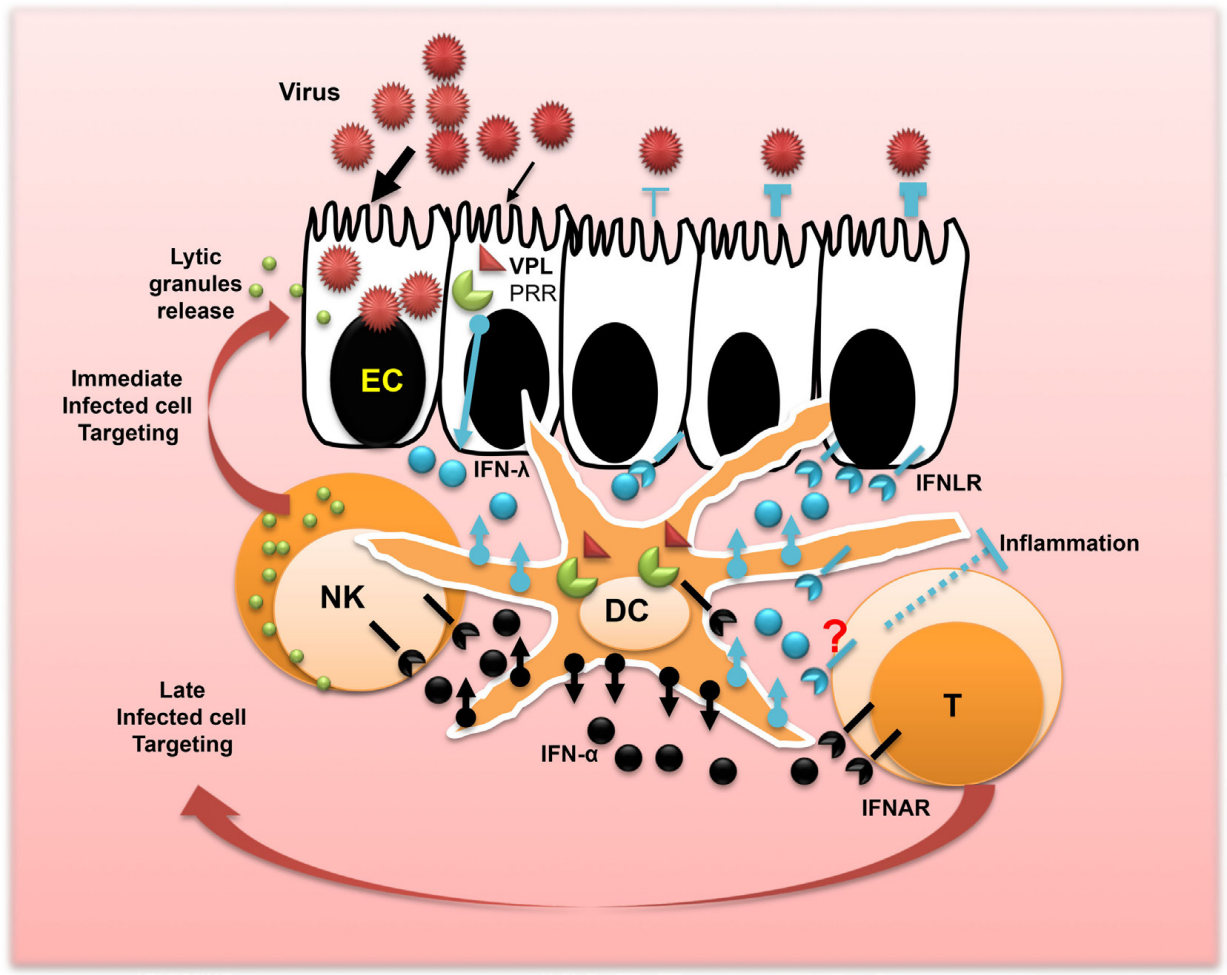
**Concerted action of IFN-λ and IFN-α promotes mucosal immunity and viral protection**. After the interaction of the epithelium surface of the mucosa with viruses, epithelial cells release IFN-λ. The process leading to IFN-λ production is triggered by the interaction of a viral particle ligand (VPL) such as DNA or RNA with the cellular pattern recognition receptors. Dendritic cells (DCs) are the main immune cells of the mucosa involved in sensing viral infections and producing high amounts of IFN-λ and IFN-α. This produced IFN-λ fuels the antiviral protection of the epithelium and in concert with IFN-α may shape local immunity and control inflammation. Released IFN-α by DCs also plays a central role in controlling viral dissemination in the lamina propria and the promotion of T cell immunity and natural killer cell activation for immediate targeting and clearance of infected cells toward stopping viral spread.

In contrast to type I IFN, type III IFN was not involved in controlling viral infection of lamina propria in agreement with the lack of response of effector immune cells to IFN-λ but not to IFN-α (Figure 2). Therefore, type I and type III IFNs are not redundant cytokines at least in the GI tract (25, 45).

Regarding the role of IFN-λ during rotavirus infection, divergent results between virus strains and research groups have been reported. Early studies showed that IFN-λ is highly effective in controlling the murine rotavirus infection in suckling mice (27, 43). However, Lin et al. demonstrated that the effect of IFN-λ is dependent on the rotavirus strain used (46, 47). IFN-λ was able to control rotavirus infection when suckling mice received the heterologous but not the homologous rotavirus. Although the homologous rotaviruses used by those different groups are quite similar, the IFN-λR1 knock-out mice used in the more recent study were generated differently. In this study, only exon 3 of *IFN-*λ*R1* gene is lacking, while in the transgenic mice used by the other groups, the entire *IFN-*λ*R1* gene is missing. Although the first generated IFN-λR1-deficient mice have been extensively studied by many groups for almost a decade (48), comparison between the two IFN-λR1-deficient mice is warranted. Important standardizations of virus dosage, virus strain, host strain, and diet are also required for minimizing the variance in the experimentation.

## Role of IFN-λ in the Control of Vaginal Mucosa Immunity

Herpes simplex virus particularly 2 (HSV-2) is the prevalent cause of genital ulceration in humans worldwide with lifelong latent infection of female genital mucosa (49). Early studies in mice using a model of localized HSV-2 infection demonstrate that IFN-λ completely prevents virus replication in the vagina. IFN-λ has been shown to induce significant virus suppression associated with a complete remission from the genital viral disease. Antiviral effects of IFN-λ were superior in comparison with IFN-α (48, 50, 51). In a recent study using BAC transgenic mice, expressing firefly luciferase under transcriptional control of the Mx2 gene promoter, it has been also demonstrated that IFN-λ reactivity was most prominent in mucosal surfaces, including the genital area; however, IFN-α response was strong in the liver, spleen, and kidney (52). Furthermore, the vaginal mucosa expresses high levels of IFN-λ. In contrast to IFN-α, NF-κB plays a major role in promoting IFN-λ expression (53). The primary source of IFN-λ was attributed to dendritic cells (DCs), indicating that IFN-λ response plays a crucial role in promoting vaginal mucosa immunity (Figure 2). Ongoing investigations, using IFN-λ transgenic models, will likely determine the effector cells responsible for the control of vaginal infection by IFN-λ.

## Perspectives and Challenges of IFN-λ as a New Player in Mucosal Surfaces

The role of IFN-λ in controlling viral infection of mucosal surfaces is increasingly studied (5, 10). The interaction between IFN-λ and the EC of the mucosa is a crucial step in establishing this antiviral protection. In comparison with IFN-α, the antiviral effect of IFN-λ on the lining epithelium appears significantly superior. However, IFN-λ fails to control viral infection of immune areas underlying the epithelium. Conversely, IFN-α appears more efficacious than IFN-λ in protecting immune tissues and lamina propria from viral infection. By playing complementary roles, both IFN-λ and IFN-α seem instrumental in protecting mucosal surfaces from viruses. However, the role of immune cells that contributes to the antiviral activity of IFN-λ and IFN-α remains poorly understood. Besides DCs, we still poorly understand the contribution of other immune system components in IFN-λ functions within the mucosa. It has been clearly demonstrated that the mean source of IFN-λ in the mucosa is coming from the DCs. This released IFN may fuel the antiviral protection of EC, and probably in concertation with IFN-α, modulates mucosal immunity and inflammation (Figure 2). However, studies in this field concern mostly IFN-α. The role of IFN-α in activating immune cells during viral infection of the mucosa and related inflammation has been relatively well studied (10). The question yet to be answered is, in the context of IFN-α and viral infections, what is the role of IFN-λ in innate immunity and inflammation, particularly in neonates and infants?

Due to their innate immune deficiency, neonates and infants are highly sensitive to respiratory and GI virus infections leading to high risk of mortality (54, 55). Immaturity of natural killer (NK) cells has been demonstrated as the primary factor for increased susceptibility to viral infections in early life for both human and mice (56–59). NK cell responses’ impairments are associated with a significant deficiency in the production and the release of lytic granules (60), in agreement with early studies demonstrating that the transfer of adult NK cells to suckling mice induces a protection against viral infection (61). This conclusion has been corroborated by a recent report demonstrating that NK cell deficit can be reversed in suckling mice (62). In parallel, as reported earlier, it has been shown that IFN-λ plays a crucial role in viral infections of suckling mice (27, 43, 46, 47), suggesting that IFN-λ may induce antiviral functions at least partially *via* NK cells, and those functions of IFN-λ are missing in neonates and infants due to a potential deficiency of IFN-λ production or response. The aptitude of NK cells to respond rapidly without prior sensitization makes them at the front line of defense against infection (63, 64). NK cells are well armed for sensing and killing virus-infected cells (Figure 2). *In vivo* activation of NK cells by IFN-λ has been well documented in cancer models (6, 65). Significant NK cell impairment of NK cell tumoricidal activity has been reported in IFN-λR−/− mice (66). More recently, we have demonstrated that a cooperation between IFN-λ and IFN-α promoted local NK cell antitumor actions (67). We believe that within mucosal surfaces, IFN-λ in combination with IFN-α may play an important role in recruiting and activating NK cells to clear viral infections. Privileged interaction of IFN-λ with EC may not only induce the antiviral state but also contribute to the attraction of immune cells *via* the release of potential chemokines.

In addition to its role in mucosal immunity and viral infections, IFN-λ has been recently proposed as the treatment of choice for IAV infection because its antiviral activity was not associated with an exacerbation of inflammation in contrast to IFN-α (32). However, the mechanisms leading to the potential anti-inflammatory role of IFN-λ remain elusive. In the case of IAV infection, it has been indicated that IFN-λ was simply acting on EC without overstimulating the immune system and driving immunopathology like IFN-α. However in collagen-induced arthritis, the anti-inflammatory role of IFN-λ has been demonstrated (68). Apparently, IFN-λ decreased significantly neutrophil population in the joints of diseased mice. This occurred in association with a reduction of interleukin-1β level, which is thought to play a crucial role in inflammation.

## Conclusion

We currently see a clearer picture about the role of IFN-λ and its possible therapeutic uses. All studies highlight the crucial role of IFN-λ on EC, which are the first line of attack by pathogens, toxins, and other damaging agents. The majority of infections and cancers concern epithelial cell types. This strongly suggests that beyond its well-described antiviral and antitumoral roles, IFN-λ may have immunomodulatory roles for indirectly protecting EC from different damages. New ideas have already emerged about the role of IFN-λ on effector cells orchestrating inflammation and autoimmunity. However, for building successful strategies against cancer and infection diseases, the interaction between IFN-λ and IFN-α should be taken into consideration. Based on new evidence from viral infections and cancer studies a concerted action of IFN-α and IFN-λ seems crucial in the complexity of interactions between diseased cells and surrounding immune cells.

## Author Contributions

AL: designed the plan of the manuscript and the figures and wrote the manuscript. AZ, AT, and KC-S: discussion on the plan and the references used in the manuscript and contribution in writing the manuscript.

## Conflict of Interest Statement

The authors declare that the research was conducted in the absence of any commercial or financial relationships that could be construed as a potential conflict of interest.
